# An assessment of health risks posed by consumption of pesticide residues in fruits and vegetables among residents in the Kampala Metropolitan Area in Uganda

**DOI:** 10.1186/s40550-022-00090-9

**Published:** 2022-04-28

**Authors:** Charles Ssemugabo, Asa Bradman, John C. Ssempebwa, Fenna Sillé, David Guwatudde

**Affiliations:** 1 Department of Disease Control and Environmental Health, School of Public Health, Makerere University College of Health Sciences, Kampala, Uganda; 2 Department of Public Health, School of Social Sciences, Humanities and Arts, University of California Merced, Merced, CA 95343 USA; 3 Center for Children’s Environmental Health Research, School of Public Health, University of California, Berkeley, CA 94704 USA; 4 Department of Environmental Health and Engineering, The Johns Hopkins University Bloomberg School of Public Health, Baltimore, MD 21205 USA; 5 Department of Epidemiology and Biostatistics, School of Public Health, Makerere University College of Health Sciences, Kampala, Uganda

**Keywords:** Maximum residual limits, Hazard quotient, Estimated daily intake, Acceptable daily intake, Uganda

## Abstract

**Background:**

Pesticide use for fruits and vegetable production in Uganda may result in presence of residues on produce which may pose health risks to consumers. Uganda does not have an established system for monitoring pesticide residues in fruits and vegetables and assessing potential health risks. This research aimed to conduct a health risk assessment of presence of pesticide residues in fruits and vegetables in the Kampala Metropolitan Area in Uganda.

**Method:**

Pesticides were measured in 160 fruits and vegetables samples collected at farms, markets, street vendors, restaurants and homes; and analysed using liquid chromatography-tandem mass spectrometry and gas chromatography-mass spectrometry. Fruit and vegetable consumption information was collected from 2177 people. Pesticide concentrations were compared with European Union maximum residual limits (MRLs). Mean values of pesticide concentration residues found in the sample of fruits and vegetables; and fruits and vegetables intake and body weight were used to calculate the estimated daily intake (EDI) of pesticide residues. EDI values were compared with acceptable daily intakes (ADI) to calculate the hazard quotient by age group, and stage at which consumption happens along the chain.

**Results:**

Overall, 57 pesticides were detected in fruits and vegetables from farm to fork. Of the 57, 39 pesticides were detected in all the fruits and vegetables studied. Concentrations of fonofos, fenitrothion and fenhexamid were above the European Union MRLs in some samples. Hazard quotients based on dietary ingestion scenarios for 18 pesticides, including dichlorvos (444) alanycarb (314), fonofos (68), fenitrothion (62), dioxacarb (55) and benfuracarb (24) and others, were above 1, indicating the possibility of chronic health risk to consumers. Chronic health risk decreased with age but was stable for stage at which consumption happens along the food chain. The number of pesticides with EDI greater than the ADI decreased with increase in age; with 18, 13, 9, 11, 8, 9, and 9 pesticides for age groups < 5, 5-12, 13-19, 20-25, 36-49 and ≥ 50 respectively.

**Conclusion:**

Chronic dietary pesticide exposures to Ugandans are likely common, and for some pesticides result in exposure exceeding health-based benchmarks. Risks were highest for younger participants. There is an urgent need to increase monitoring and regulation of pesticides in fruits and vegetables in order to protect consumers, especially the children who are vulnerable to the adverse effects of pesticides.

**Supplementary Information:**

The online version contains supplementary material available at 10.1186/s40550-022-00090-9.

## Introduction

Pesticides are widely used in agriculture to control pests and disease in crops to improve the quality of produce (Aktar et al. 2009). Some commonly used classes of pesticides include organophosphates, carbamates, pyrethroids and neonicotinoids (Matowo et al. 2020; Maggi et al. 2019; Fuhrimann et al. 2021; Staudacher et al. 2020). Most of these pesticide chemical groups, such as organophosphates, are broad spectrum insecticides, fungicides or herbicides used to control many different pests, diseases or weeds in different crops (Hill et al. 2017). Many organophosphates, carbamates, pyrethroids and neonicotinoids, all neurotoxic pesticides, are registered for use in Uganda, (Ministry of Agriculture Animal Industry and Fisheries, 2018) and use is increasing with increasing consumption of fruits and vegetables including tomatoes, cabbage and watermelons, to name but a few (Ngabirano and Birungi 2020).

Organophosphates and carbamates pesticides are generally not persistent because they degrade when exposed to sunlight, air and soils, but they often have high solubility and volatility and are heavily used in many farming systems (Akkad and Schwack 2010). Organophosphates and carbamates inhibit cholinesterase and may impact neurodevelopment by other mechanisms, including interference in synaptogenesis and myelin sheath formation (Vale and Lotti 2015; Sagiv et al. 2019). Pyrethroids and neonicotinoids are often systemic pesticides with a higher affinity to soil and, especially for neonicotinoids, have the potential to bioaccumulate. They also have low volatility (Laskowski 2002; Bonmatin et al. 2015). Pyrethroids act by altering the function of voltage-gated sodium channel and consequently disrupt electrical signalling in the nervous system (Soderlund 2010) and are generally less acutely toxic than organophosphates (Simaremare et al. 2019). However, they are neurotoxicants and have been associated with confusion, lacrimation and salivation (Bradberry et al. 2005) and also poorer development and asthma in children (Pitzer et al. 2021; Vester et al. 2019). The mechanism of toxicity for neonicotinoids is based on selective binding and interaction with nicotinic acetylcholine receptor sites of a target organism causing paralysis that leads to death (Taillebois et al. 2018; Cartereau et al. 2021; Houchat et al. 2020), and they have also been associated with development or neurological disorders (Cimino et al. 2017) in humans.

The use of these chemicals in agriculture may result in residues in food and expose consumers. Events where high levels of pesticide contamination has occurred have resulted in acute health risks including nausea, excessive sweating and salivation, diarrhoea and vomiting, inhibition of blood clotting, and paralysis of the respiratory and circulatory systems (PAN 2018). Several studies have shown that chronic exposure to low levels of some neurotoxic pesticides are associated with poorer learning and behavioral problems in children, memory loss, loss of coordination, reduced speed of response to stimuli, reduced visual ability, altered or uncontrolled mood and general weakness; reproductive defects and cancers (Nicolopoulou-Stamati et al. 2016; Coker et al. 2018; Chiu et al. 2018).

In Uganda, the volume of pesticides used has increased from 338 t in the 1960s to 18,928.16 t in 2019 (FOA: FAOSTAT 2021). Many farmers do not follow recommended mixing concentrations on label instructions and pre-harvest intervals (Kaye et al. 2015). Such improper pesticide use practices may result in higher levels of pesticide residues in fruits and vegetables (Grewel et al. 2017) that leave the farm to the final consumer. While they are important sources of minerals, vitamins, and other healthful nutrients, consumption of fruits and vegetables contaminated with pesticide can be a route of exposure to hazardous chemicals. Fruit and vegetable consumption is a protective factor for noncommunicable diseases such as diabetes (World Health Organisation 2013), and consumption is rising among Ugandans, which consume an average 260 g of fruits and vegetables each day (Ssemugabo et al. 2021a). Fruit and vegetable consumption has grown among residents of the Kampala Metropolitan Area (KMA) (Kabwama et al. 2019), and organophosphate, carbamate, pyrethroid and neonicotinoid pesticides have been previously detected in the tomatoes, watermelon, cabbages among others in this market (Kaye et al. 2015; Ssemugabo et al. 2021b; Atuhaire et al. 2017).

In the current study, we assessed potential pesticide exposures and health risks from consumption of fruit and vegetables by residents of the KMA, in Uganda.

## Materials and methods

### Study area

This study was conducted in Kampala, Wakiso and Mukono Districts, three of the 5 districts that make up the KMA in Uganda. The 3 districts have a population of 10,812,700 people (UBOS 2018) and cover an area of 1000 km^2^ (Kasimbazi 2016). Agriculture is the largest economic activity in Central Uganda within which the KMA is located, supporting 39.3% of the population (UBOS 2018). This region has many large fresh produce markets, restaurants, fruit and vegetable vending along the streets, as well as many of the farms where fruits and vegetables consumed within central Uganda are grown. Kampala, Wakiso and Mukono are inhabited by 15% of Uganda’s population and contain Uganda’s districts that consume a large volume of the fruit and vegetables produced.

Ethical clearance to conduct the study was obtained from the Makerere University School of Public Health Higher Degrees, Research and Ethics Committee; and registered by Uganda National Council for Science and Technology (SS 5203). Participation in the study was voluntary and participants (farmers, restaurants market managers, street fruit and vegetable vendors, and household heads) provided informed written consent to collect samples and fruit and vegetable dietary intake information. All samples and questionnaire were coded with an anonymous identification number.

### Pesticide residue data

#### Sampling of fruits and vegetables

Fruits and vegetables samples were collected from key stages along the supply chain including farms (50), markets (50), street vendors (20), restaurants (20) and homes (20), totaling 160 samples. The detailed methodology used to collect the fruits and vegetable samples has been previously described (Ssemugabo et al. 2021b). Briefly, fresh fruit and vegetable samples were purchased and collected in sterile polythene bags or PET (polyethylene terephthalate) plastic containers from selected farms, markets, and street vendors. Samples of ready-to-eat foods were bought from restaurants and homes, especially juices and salads that do not contain fat-soluble substances. Three replicate fruit and vegetable samples were collected at each location measuring at least 1 kg for small and 2 kg for large produce as suggested by Codex guidelines (El-Zaher et al. 2011; Food and Agriculture Organisation 1999); processed food samples were at least 1 kg or 1 l in case of juice. The samples were stored in a cooler and transported to the laboratory within 8 h and stored at − 20 °C until analysis.

#### Sample preparation and extraction

A total of 93 pesticides residues were screened in the fruit and vegetable samples (Supplementary Table 1). Using the Quick, Easy, Cheap, Effective, Rugged and Safe (QuEChERS) approach, samples were prepared, cleaned and extracted to determine of pesticide residues (Anastassiades et al. 2002). Briefly, 1-2 kgs of fruit or vegetable was chopped, grinded and blended to homogenize the sample. Of the homogenized sample, 200 g was put into containers and immediately frozen in order to minimize the risk of degradation of any pesticide residues present. Ten grams of homogenized sample was mixed with 3 g of sodium bicarbonate (NaHCO3) and 20.0 mL acetonitrile, vortexed and placed on a mechanical shaker at 300 rpm/min for 15 min to improve extractability of pesticide residues and then centrifuged for 3 min at 3200 rpm. To this, 10 g of anhydrous sodium sulphate (Na2SO4) was then added, vortexed and centrifuged for 3 min at 3200 rpm. We filtered the crude extract using a 0.2-μm PTFE syringe filter. The final supernatant layer (0.50 g /mL) was transferred into the vials and injected into the LC-MS/MS for analysis of pesticide residues (Ssemugabo et al. 2021b).

#### Pesticide analysis

Liquid chromatography – Tandem mass spectrometry (LC-MS/MS) analysis was carried to detect and ensure quality of the pesticides residue measurements. A zorbax eclipse plus C18 capillary column (150 mm with 2.1 mm internal diameter and 1.8 μm particle size) operating at 35 °C to 360 °C was used with the internal temperature set at 35 °C for 1 min, then ramped to 120 °C per minute and 375 °C per minute. This process was run over two mobile phases. Phase A involved – water (0.1% formic acid, 5 mM ammonium formate, and 2% MeOH). Phase B involved – methanol (0.1% formic acid, 5 mM ammonium formate, 2% water). The injector temperature was 120 °C and carrier gas was helium at a flow rate of 13 L/minute with splitless injection. The injection volume was 5 μL at a pressure of 45 psi. The MS ion source temperature was 120 °C for a minute and raised at a rate of 35 °C per minute to 375 °C. Confirmation analysis utilised LC-MS/MS which requires two product ions. Compounds with only one product ion were quantified and confirmed using the second ion. For confirmation, the relative ion intensity for a pesticide in a sample was calculated and the value compared to the equivalence for a calibration standard. For positive confirmation, the retention times were matched to the calibration standard as well the relative ion intensities according to the recommended maximum tolerances. Limits of detection (LOD) was determined during the method validation and measurements of uncertainty.

The method developed by Keppel et al. at the United States Food and Drug Administration (U.S. FDA) (Kabwama et al. 2019; Ssemugabo et al. 2021b) was used to measure dithiocarbamates (mancozeb, maneb, dithane, thiram, metam sodium and propineb. Frozen sub-samples of 10 g were placed into a Duran bottle (250 ml) and mixed with isooctane (20 ml) followed by stannous chloride (reducing solution) in hydrochloric acid (100 ml), and sealed immediately with a septum and cap. The sample was incubated at 80 °C in a water bath for 1.5 h with frequent shaking. The Duran bottles were removed and left at ambient temperature for approximately 1 h. The bottles were frozen for 30 min to allow the generated carbon disulphide gas to condense. The samples were shaken and left for 5 min. The organic phase (iso-octane) was removed and placed in a vial prior to the quantitation of carbon disulphide by Gas Chromatography-Mass spectrometry (GC–MS). Spiking was done twice, once at the limit of quantitation (LOQ) (50 μg/kg) and another at the expected residue level (1000 μg/kg), as obtained from previous runs during instrument optimization (mean recoveries for individual pesticides in the range 60 – 140%) and precision (RSD_r_ ≤ 12%). A 5-point calibration was used, ranging from 0.125–5 μg/ml. The method’s LOQ was set at 0.05 mg/kg which equates to the calibration standard of 0.125 μg/ml. All extracts were analyzed using GC-MS. Final pesticide residues concentrations were expressed in mg/kg of food.

### Dietary consumption data

A modified semi-structured food frequency questionnaire from the World Health Organization’s (WHO) STEPwise approach to surveillance, standardized method of collecting data on risk factors for noncommunicable diseases (NCDs) (WHO 2017) was used was used to interview 2177 participants to assess fruit and vegetable consumption over a 24-h dietary recall period and their body weight was concurrently measured using a weighing scale. The detailed methodology on this has been described elsewhere (Ssemugabo et al. 2021a). Briefly, based on typical Ugandan diets, a food album was developed with different quantities of selected fruits and vegetables. Each research assistant was given a copy of the food album as a guide during the interview. Respondents were asked to identify the quantities they consume per serving to determine the amounts consumed. Based on portion size in the food album, we estimated intake in grams of each fruit and vegetable each day of the week. For children under 18 years, their parents or caretakers were interviewed. Participant’s weight was also measured thrice and the average calculated. For children below 2 years who cannot stand, their weight was obtained by reviewing their immunization chart or asking their parents or caregivers the measurement from their last weighing event. Socio-demographics data was also obtained using the study questionnaire. Consumption data was collected for five commonly consumed and pesticide intensive fruits and vegetables, that is: watermelon, passion fruit, tomato, cabbage and eggplant following interviews with farmers and agricultural extension. Workers.

### Health risk assessment

We first prepared descriptive statistics of the pesticide residue levels in the produce samples. The mean pesticide concentrations were then compared with European Union maximum residual limits (EU MRLs) obtained from the pesticide residue database (https://ec.europa.eu/food/plant/pesticides/eu-pesticides-database/mrls/?event=search.pr) (EUROPEAN UNION 2021). EU MRLs were used because they provided comprehensive standard values for all fruits studied; they have also been used in other African studies (Fosu et al. 2017; Issa et al. 2018). We also used the mean pesticide concentrations to calculate the estimated pesticide intake to compare with acceptable daily intakes (ADIs). Estimated daily intake (EDI) (mg/kg/bw/day) was calculated by multiplying the mean concentration of each pesticide (C) and the fruits and vegetable consumption rate (FVCR) (g/day) and dividing this by body weight (BW) using the following formula EDI = (C x FVCR)/BW (Gad Alla et al. 2015; Chen et al. 2011). The FVCR used was obtained from the dietary consumption survey (Ssemugabo et al. 2021a). FVCR was calculated as mean consumption of fruits and vegetables studied for the sample population as well as for different age groups studied that is < 5, 5-12, 13-19, 20-24, 25-35, 36-49 and 50+. BW used was measured during the dietary consumption survey with the mean for general sample population and age groups calculated accordingly. The ADI (mg/kg/bw/day) for the different pesticides was obtained from the EU pesticide residue database (EUROPEAN UNION 2021). The chronic risk assessment for pesticide residue was calculated by comparing EDI with the ADI to get the hazard quotient (HQ) using the following equation; HQ = EDI/ADI. A hazard quotient (HQ) > 1 indicates exposures over the health-based benchmarks and the potential to induce unacceptable health risks among consumers.

## Results

### Pesticide residue concentrations

The mean concentration of organophosphates, carbamates, pyrethroids and neonicotinoids among other pesticides detected in watermelons, passion fruit, tomato, cabbage and eggplants and comparisons with their respective MRLs are shown in Table 1. Out of the 62 pesticide active ingredients detected, 5 were excluded due to the lack of verified maximum residue levels (MRLs) in the EU database for the studied fruits and vegetables. Therefore, 57 pesticides were considered for the risk assessment. Of the 57 pesticides, 39 pesticides were detected in all the fruits and vegetable types. Dimethoate was detected only in watermelon with a mean concentration of 0.0007 mg/kg. Fonofos was detected in all fruits and vegetables with concentrations above the MRLs in watermelon (0.03 mgkg), passion fruit (0.02 mg/kg), cabbages (0.11 mg/kg) and eggplants (0.14 mg/kg). Methidathion was not detected in watermelon and cabbages and malathion was not detected in passion fruit. Methacrifos was detected in passion fruit at 0.00003 mg/kg and cabbages at 0.000002 mg/kg. Ethoprophos was not detected in vegetables but only in eggplants at 0.0003 mg/kg. Coumaphos and pirimiphos-methyl were detected only cabbages at 0.0000005 mg/kg and eggplants at 0.00001 mg/kg respectively. Apart from passion fruit, fenitrothion concentration was above the MRLs in watermelon (0.02 mg/kg), tomato (0.013 mg/kg), cabbage (0.03 mg/kg) and eggplant (0.03 mg/kg). Neonicotinoids were detected in almost all fruits and vegetables apart from thiacloprid that was only detected in passion fruit 0.000007 mg/kg and tomato 0.000002 mg/kg. Deltamethrin, azoxystrobin and proquinazid were only detected in vegetables with concentrations below the MRLs. Although not detected in tomato, fenhexamid’s concentration was above the MRLs in watermelon (0.01 mg/kg), passion fruit (0.07 mg/kg) and cabbage (0.03 mg/kg).

**Table 1:** Concentration of pesticide residues per fruit and vegetable type compared with the MRLs

Pesticide residues	LOD (mg/kg)	Water melon (mg/kg)		Passion fruit (mg/kg)		Tomato (mg/kg)		Cabbage (mg/kg)		Eggplant (mg/kg)	
		Mean	MRL	Mean	MRL	Mean	MRL	Mean	MRL	Mean	MRL
Dithiocarbamate^a^	0.000006	0.001	1.5	0.00007	0.05	0.0002	3	0.0006	3	0.0004	3
Omethoate	0.00002	0.0004	0.01	0.0002	0.01	0.0003	0.01	BDL	0.01	0.00007	0.01
Acephate	0.00003	0.001	0.01	0.001	0.01	0.0001	0.01	0.00008	0.01	0.0002	0.01
Monocrotophos	0.00001	0.00003	0.01	0.00004	0.01	BDL	0.01	0.00003	0.01	0.00002	0.01
Vamidothion	0.00001	BDL	0.01	BDL	0.01	BDL	0.01	BDL	0.01	0.00008	0.01
Dimethoate	0.000008	0.0007	0.01	BDL	0.01	BDL	0.01	BDL	0.01	BDL	0.01
Mevinphos	0.00003	BDL	0.01	BDL	0.01	BDL	0.01	0.00004	0.01	0.00005	0.01
Phosphamidon	0.00002	BDL	0.01	BDL	0.01	BDL	0.01	BDL	0.01	0.00005	0.01
Fonofos	0.00001	**0.03**^**a**^	**0.01**	**0.2**^**a**^	**0.01**	0.2	10	**0.1**^**a**^	**0.01**	**0.1**^**a**^	**0.01**
Azamethiphos	0.000005	0.00002	0.01	0.000007	0.01	BDL	0.01	BDL	0.01	BDL	0.01
Dichlorvos	0.00002	0.003	0.01	0.007	0.01	0.0001	0.01	0.0002	0.01	0.0007	0.01
Malaoxon	0.00001	BDL	0.02	BDL	0.02	0.00004	0.02	0.00002	0.02	BDL	0.02
Methidathion	0.00001	BDL	0.02	0.000003	0.02	0.00002	0.02	BDL	0.02	0.000001	0.02
Malathion	0.00002	BDL	0.02	BDL	0.02	BDL	0.02	BDL	0.02	0.00007	0.02
Methacrifos	0.000005	BDL	0.01	0.00003	0.01	BDL	0.01	BDL	0.01	BDL	0.01
Ethoprophos	0.00008	BDL	0.01	BDL	0.01	BDL	0.01	BDL	0.01	BDL	0.01
Fenamiphos	0.000009	BDL	0.02	BDL	0.02	BDL	0.04	BDL	0.04	BDL	0.02
Quinalphos	0.00003	0.0001	0.01	0.0001	0.01	0.00003	0.01	BDL	0.01	0.0001	0.01
Coumaphos	0.00002	BDL	0.01	BDL	0.01	BDL	0.01	BDL	0.01	BDL	0.01
Chlorpyriphos-methyl	0.000008	BDL	0.01	0.00004	0.01	0.00009	0.01	0.00005	0.01	0.00002	0.01
Temephos	0.000008	BDL	0.01	0.00001	0.01	BDL	0.01	0.000009	0.01	BDL	0.01
Profenofos	0.00001	0.003	0.01	0.00002	0.01	0.04	10	0.003	0.01	0.005	0.01
Pirimiphosmethyl	0.00002	BDL	0.01	BDL	0.01	BDL	0.01	BDL	0.01	0.00001	0.01
Fenitrothion	0.00001	**0.02**^**a**^	**0.01**	0.004	0.01	**0.01**^**a**^	**0.01**	**0.03**^**a**^	**0.01**	**0.03**^**a**^	**0.01**
Aminocarb	0.00002	BDL	0.01	0.0007	0.01	BDL	0.01	0.00008	0.01	0.00002	0.01
Methomyl	0.00003	0.00006	0.015	0.00003	0.01	0.00003	0.01	BDL	0.01	0.00003	0.01
Aldicarbfragment	0.00001	0.00002	0.02	0.00002	0.02	0.00004	0.02	0.00002	0.02	BDL	0.02
Pirimicarb	0.00003	0.00004	0.5	0.00004	0.01	BDL	0.5	BDL	0.5	BDL	0.5
Dioxacarb	0.00001	0.004	0.01	0.003	0.01	0.003	0.01	0.003	0.01	0.004	0.01
Carbaryl	0.000008	BDL	0.01	BDL	0.01	BDL	0.01	BDL	0.01	0.00001	0.01
Carbofuran	0.000009	0.00003	0.01	BDL	0.01	BDL	0.002	BDL	0.002	0.00003	0.002
Alanycarb	0.00001	0.0001	0.02	0.00006	0.02	0.08	0.02	0.01	0.02	0.006	0.02
Benfuracarb	0.00005	0.0005	0.01	BDL	0.01	0.004	0.002	0.07	0.002	0.004	0.002
Methiocarb	0.00004	BDL	0.03	BDL	0.03	BDL	0.03	0.00005	0.03	BDL	0.03
Imidacloprid	0.00003	0.0007	0.2	0.0008	0.05	0.0004	0.5	0.0004	0.5	0.0002	0.5
Acetamiprid	0.00002	0.004	0.2	0.002	0.01	0.008	0.5	0.005	0.4	0.001	0.2
Thiacloprid	0.00001	BDL	0.2	BDL	0.01	BDL	0.5	BDL	0.3	BDL	0.7
Bifenthrin	0.00002	0.0001	0.01	0.00004	0.01	0.0004	0.3	0.00005	0.4	BDL	0.3
Lambda-Cyhalothrin	0.00002	0.0002	0.06	0.0001	0.01	0.0002	0.07	0.0002	0.15	0.0002	0.3
Deltamethrin	0.00001	BDL	0.02	BDL	0.01	BDL	0.07	0.00006	0.1	BDL	0.4
Cypermethrin	0.00001	0.0002	0.2	BDL	0.05	0.001	0.5	0.0004	1	0.0004	0.5
Carbendazim	0.00002	BDL	0.1	0.0001	0.1	BDL	0.3	0.0001	0.1	BDL	0.5
Imazalil	0.00001	0.0005	0.01	0.0004	0.01	0.0001	0.3	0.0003	0.01	0.0003	0.01
Metazachlor	0.00001	0.00001	0.02	0.00004	0.02	0.00006	0.02	0.00002	0.4	0.00002	0.02
Metalaxyl	0.00002	BDL	0.2	BDL	0.01	0.00005	0.3	BDL	0.06	BDL	0.01
Azaconazole	0.000006	0.000009	0.01	0.0001	0.01	0.000008	0.01	0.00007	0.01	0.000008	0.01
Clomazone	0.000007	BDL	0.01	BDL	0.01	BDL	0.01	BDL	0.01	BDL	0.01
Azoxystrobin	0.000007	BDL	1	BDL	4	0.00456	3	0.003	5	0.003	3
Pyrimethanil	0.00002	0.0001	0.01	0.00008	0.01	0.0001	1	0.00008	0.01	0.00006	1
Spirotetramat	0.00002	0.00003	0.2	BDL	0.1	0.00009	2	0.00001	2	BDL	2
Fenhexamid	0.00001	**0.01**^**a**^	**0.01**	**0.07**^**a**^	**0.01**	BDL	2	**0.03**^**a**^	**0.01**	0.009	2
Fenarimol	0.00001	0.0006	0.05	0.0003	0.02	0.0002	0.02	0.0003	0.02	0.0004	0.02
Fluazifop	0.00002	0.005	0.01	BDL	0.01	BDL	0.06	0.0004	0.01	BDL	1
Flufenoxuron	0.00002	BDL	0.01	BDL	0.01	BDL	0.01	BDL	0.01	BDL	0.01
Pyriproxyfen	0.000007	BDL	0.05	BDL	0.05	BDL	1	BDL	0.05	BDL	1
Quinoxyfen	0.00003	BDL	0.05	0.00005	0.02	0.00004	0.02	BDL	0.02	0.00003	0.02
Proquinazid	0.00001	BDL	0.02	BDL	0.02	0.001	0.15	0.0003	0.02	0.00009	0.02

*BDL* Below detection limits, *LOD* Limit of Detection

^a^Above the MRLs

### Health risk assessment by stage of consumption along the chain

**Fig. 1:**
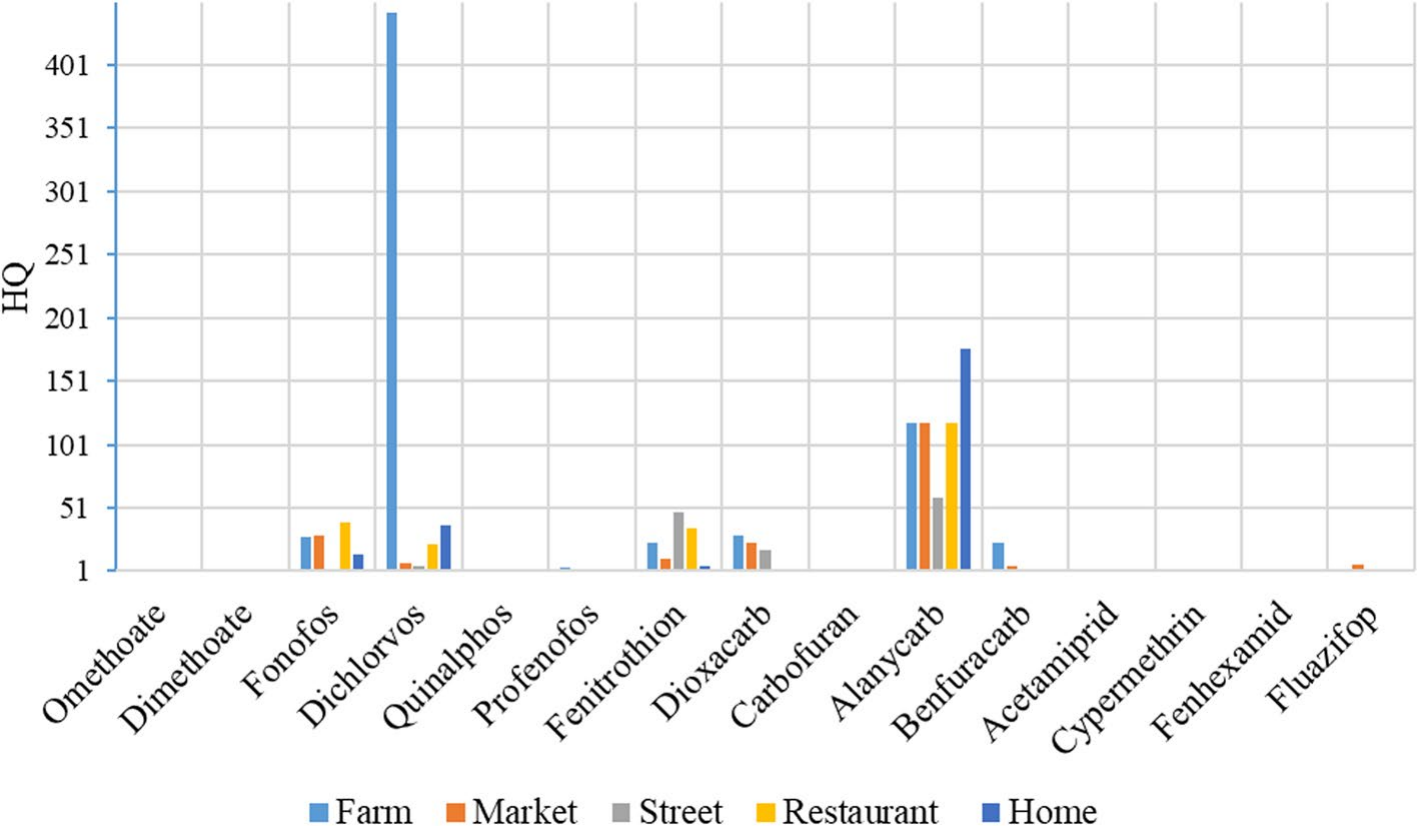
Hazard quotients for various pesticide residuals, for fruits and vegetables by stage of consumption along the chain

The risk of exposures to pesticides residues in fruits and vegetables are evaluated by the stage at which consumption may occur along the chain including at the farm, market, street vendor, restaurant and home as shown in Table 2. The EDI was higher than the ADI in at least one of the stages at which consumption may occur in 16 of the 57 pesticides assessed. EDIs for dichlorvos, fenitrothion, alanycarb and benfuracarb were above the ADI at all stages of consumption. EDIs for fonofos and profenofos exceeded the ADI at four stages of consumption. Fonofos, dichlorvos, fenitrothion, dioxacarb, alanycarb and benfuracarb presented the highest risk levels with HQs of 27.5, 442.6, 23.6, 29.5, 118.0 and 23.6 respectively, at the farm and throughout the entire supply chain (See supplementary Table 2). Overall, pesticide concentration at street vendors presented lower HQs and consequently lower likelihood for health risks compared to other stages along the chain (Fig. 1).

**Table 2:** Estimated daily intake (mgkg/bw/day) for fruits and vegetables by stage along the chain

Pesticides	ADI (mg/kg/bw/day)	EDI (mg/kg/bw/day)				
		Farm	Market	Street	Restaurant	Home
Dithiocarbamate^a^	0.05	0.002	0.003	0.005	0.002	0.002
Omethoate	0.002	4.7E-06	**0.002**^**a**^	BDL	**0.002**^**a**^	2.9E-06
Acephate	0.03	0.005	0.004	0.0004	0.002	0.0006
Monocrotophos	0.0006	0.0001	0.0002	0.0002	0.0002	0.0001
Vamidothion	0.008	0.0002	0.0002	5.9E-07	3.54E-06	2.95E-06
Dimethoate	0.002	BDL	**0.003**^**a**^	BDL	BDL	BDL
Mevinphos	0.001	0.0002	0.0002	2.4E-06	2.4E-06	5.9E-05
Phosphamidon	0.0005	1.2E-06	1.18E-06	1.18E-06	BDL	1.77E-06
Fonofos	0.03	**0.8**^**a**^	**0.9**^**a**^	**0.006**	**1.2**^**a**^	**0.4**^**a**^
Azamethiphos	0.025	2.4E-06	5.9E-05	1.2E-06	5.9E-06	BDL
Dichlorvos	0.00008	**0.04**^**a**^	**0.0006**^**a**^	**0.0004**^**a**^	**0.002**^**a**^	**0.003**^**a**^
Malaoxon	0.03	4.1E-06	4.1E-06	0.0003	5.9E-05	5.9E-05
Methidathion	0.001	BDL	BDL	1.2E-06	BDL	0.0002
Malathion	0.03	0.0003	BDL	BDL	BDL	2.9E-06
Methacrifos	0.006	BDL	0.0001	BDL	BDL	BDL
Ethoprophos	0.0004	BDL	0.0001	BDL	0.0001	0.0002
Fenamiphos	0.0008	5.9E-08	1.2E-06	5.9E-05	1.8E-06	BDL
Quinalphos	0.001	0.0001	0.0005	0.0004	**0.001**^**a**^	0.0006
Coumaphos	0.001	1.8E-07	BDL	BDL	BDL	BDL
Chlorpyriphos-methyl	0.01	0.0005	BDL	2.9E-06	0.0006	BDL
Temephos	0.001	BDL	3.5E-06	BDL	2.9E-06	0.0001
Profenofos	0.03	**0.1**^**a**^	**0.04**^**a**^	**0.04**^**a**^	**0.06**^**a**^	0.004
Pirimiphosmethyl	0.03	BDL	4.7E-06	BDL	BDL	BDL
Fenitrothion	0.005	**0.1**^**a**^	**0.05**^**a**^	**0.2**^**a**^	**0.2**^**a**^	**0.02**^**a**^
Aminocarb	0.001	0.0002	**0.003**^**a**^	0.0001	0.0001	5.9E-05
Methomyl	0.0025	0.0002	0.0003	4.1E-06	0.0002	2.9E-06
Aldicarbfragment	0.001	0.0001	0.0001	0.000177	5.9E-05	0.0001
Pirimicarb	0.035	0.0001	0.0001	BDL	0.0004	5.9E-05
Dioxacarb	0.001	**0.03**^**a**^	**0.02**^**a**^	**0.02**^**a**^	BDL	BDL
Carbaryl	0.0075	2.4E-06	2.4E-06	1.8E-06	BDL	0.0001
Carbofuran	0.00015	4.1E-06	5.9E-07	**0.0003**^**a**^	**0.0002**^**a**^	2.9E-07
Alanycarb	0.001	**0.1**^**a**^	**0.1**^**a**^	**0.06**^**a**^	**0.1**^**a**^	**0.2**^**a**^
Benfuracarb	0.01	**0.2**^**a**^	**0.05**^**a**^	**0.02**^**a**^	4.7E-13	**0.02**^**a**^
Methiocarb	0.00025	0.0002	5.9E-05	0.0002	4.1E-06	5.9E-07
Imidacloprid	0.06	0.003	0.002	0.005	0.006	0.001
Acetamiprid	0.025	**0.04**^**a**^	0.02	0.01	0.01	0.005
Thiacloprid	0.01	3.5E-06	BDL	BDL	BDL	BDL
Bifenthrin	0.015	0.0006	0.002	4.7E-06	0.0002	2.4E-06
Lambda-Cyhalothrin	0.0012	0.001	0.001	0.001	0.0006	0.0006
Deltamethrin	0.01	2.4E-06	0.0002	BDL	BDL	BDL
Cypermethrin	0.0016	**0.004**^**a**^	**0.002**^**a**^	**0.005**^**a**^	0.001	0.0006
Carbendazim	0.02	1.2E-06	0.001	2.4E-06	5.9E-05	4.7E-07
Imazalil	0.025	0.002	0.001	0.004	0.002	0.002
Metazachlor	0.08	0.0002	0.0002	0.0002	0.0002	0.0001
Metalaxyl	0.08	1.2E-06	0.0002	BDL	BDL	BDL
Azaconazole	0.001	4.7E-06	0.0005	0.0004	BDL	0.0006
Clomazone	0.133	1.2E-06	BDL	BDL	BDL	BDL
Azoxystrobin	0.2	0.02	BDL	BDL	0.01	0.04
Pyrimethanil	0.17	0.0002	0.0006	0.001	0.0005	0.0006
Spirotetramat	0.05	0.0003	0.0002	0.0002	2.9E-06	0.0001
Fenhexamid	0.2	0.1	0.2	**0.6**^**a**^	0.006	0.05
Fenarimol	0.01	0.002	0.0006	0.002	0.002	0.005
Fluazifop	0.004	0.0006	**0.02**^**a**^	BDL	BDL	BDL
Flufenoxuron	0.01	1.8E-06	2.4E-06	1.8E-07	1.2E-06	4.1E-07
Pyriproxyfen	0.05	BDL	BDL	2.9E-06	BDL	BDL
Quinoxyfen	0.2	0.0002	5.9E-05	0.0003	1.2E-06	0.0003
Proquinazid	0.01	0.003	0.0006	0.002	0.001	BDL

*BDL* Below detection limit, *ADI* Acceptable Daily Intake, *EDI* Estimated Daily Intake

^a^EDI greater than ADI (HQ > 1)

### Health risk assessment by age group

**Fig. 2:**
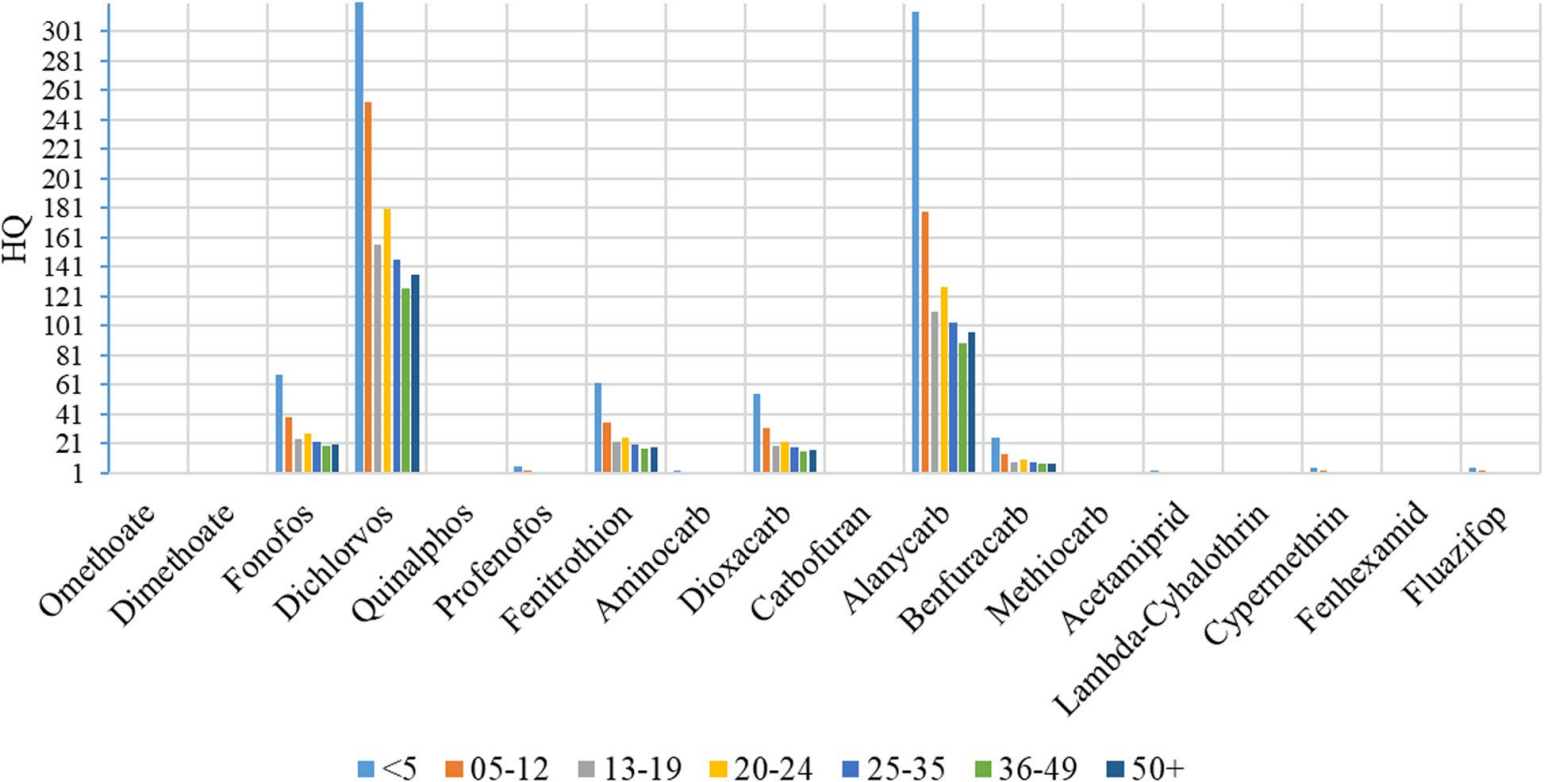
Hazard quotients for various pesticide residuals, for fruits and vegetables by age group

We evaluated the risk of consumption of pesticide residues by age of consumers as shown in Table 3. EDIs for fonofos, dichlorvos, profenofos, fenitrothion, dioxacarb, alanycarb, benfuracarb, cypermethrin and fluazifop exceeded ADIs throughout all age groups and consequently pose chronic health risks. The number of pesticides with EDIs greater than the ADI decreased with age with 18, 13, 9, 11, 8, 9, and 9 for age groups under 5 years, 5-12, 13-19, 20-25, 36-49 and 50+ years respectively. Dichlorvos had the highest risk with a HQ of 444 followed by alanycarb (314), Fonofos (68), fenitrothion (62), dioxacarb (55) and benfuracarb (24) among children under 5 with a similar trend across age groups (see supplementary Table 2). Overall, HQ values decreased across age groups with children under 5 presenting highest risks and adults 50+ having the lowest chronic health risks for the nine pesticides as shown in Fig. 2.

**Table 3:** Estimated daily intake (mgkg/bw/day) for fruits and vegetables by age group

Pesticides	ADI (mg/kg bw/day)	EDI (mgkg/bw/day)							
		General population	< 5	5-12	13-19	20-24	25-35	36-49	50+
Dithiocarbamate^a^	0.05	0.003	0.007	0.004	0.002	0.003	0.002	0.002	0.002
Omethoate	0.002	0.001	**0.003**^**a**^	0.002	0.001	0.001	0.001	0.001	0.001
Acephate	0.03	0.003	0.008	0.005	0.003	0.003	0.003	0.002	0.003
Monocrotophos	0.0006	0.0002	0.0005	0.0003	0.0004	0.0002	0.0002	0.0001	0.0002
Vamidothion	0.008	0.0001	0.0003	0.0002	0.0001	0.0001	0.0001	9.4E-05	0.0001
Dimethoate	0.002	0.0008	**0.002**^**a**^	0.001	0.0008	0.0009	0.0008	0.0007	0.0007
Mevinphos	0.001	0.0001	0.0003	0.0002	0.0001	0.0001	0.0001	9.4E-05	0.0001
Phosphamidon	0.0005	5.9E-06	1.7E-05	9.4E-06	5.8E-06	6.7E-06	5.4E-06	4.7E-06	5.1E-06
Fonofos	0.03	**0.7**^**a**^	**2.0**^**a**^	**1.2**^**a**^	**0.7**^**a**^	**0.8**^**a**^	**0.8**^**a**^	**0.6**^**a**^	**0.6**^**a**^
Azamethiphos	0.025	4.1E-05	0.0001	6.6E-05	4.1E-05	4.7E-05	3.8E-05	3.3E-05	3.5E-05
Dichlorvos	0.00008	**0.01**^**a**^	**0.04**^**a**^	**0.02**^**a**^	**0.01**^**a**^	**0.01**^**a**^	**0.01**^**a**^	**0.01**^**a**^	**0.01**^**a**^
Malaoxon	0.03	5.9E-05	0.0002	9.4E-05	5.8E-05	6.7E-05	5.4E-05	4.7E-05	5.1E-05
Methidathion	0.001	3.0E-05	8.2E-05	4.7E-05	2.9E-05	3.3E-05	2.7E-05	2.3E-05	2.5E-05
Malathion	0.03	0.0001	0.0003	0.0002	0.0001	0.0001	0.0001	9.4E-05	0.0001
Methacrifos	0.006	4.1E-05	0.0001	6.6E-05	4.1E-05	4.7E-05	3.8E-05	3.3E-05	3.5E-05
Ethoprophos	0.0004	5.9E-05	0.0002	9.4E-05	5.8E-05	6.7E-05	5.4E-05	4.7E-05	5.1E-05
Fenamiphos	0.0008	1.2E-05	3.3E-05	1.9E-05	1.2E-05	1.3E-05	1.1E-05	9.4E-06	1.0E-05
Quinalphos	0.001	0.0005	**0.001**^**a**^	0.0008	0.0005	0.0005	0.0004	0.0004	0.0004
Coumaphos	0.001	5.3E-07	1.5E-06	8.4E-07	5.2E-07	6.0E-07	4.9E-07	4.2E-07	4.5E-07
Chlorpyriphos-methyl	0.01	0.0002	0.0007	0.0004	0.0002	0.0003	0.0002	0.0002	0.0002
Temephos	0.001	3.0E-05	8.2E-05	4.7E-05	2.9E-05	3.3E-05	2.7E-05	2.3E-05	2.5E-05
Profenofos	0.03	**0.06**^**a**^	**0.2**^**a**^	**0.1**^**a**^	**0.06**^**a**^	**0.07**^**a**^	**0.06**^**a**^	**0.05**^**a**^	**0.05**^**a**^
Pirimiphosmethyl	0.03	1.8E-05	4.9E-05	2.8E-05	1.7E-05	2E-05	1.6E-05	1.4E-05	1.5E-05
Fenitrothion	0.005	**0.1**^**a**^	**0.3**^**a**^	**0.2**^**a**^	**0.1**^**a**^	**0.1**^**a**^	**0.1**^**a**^	**0.09**^**a**^	**0.1**^**a**^
Aminocarb	0.001	0.0009	**0.003**^**a**^	**0.002**^**a**^	0.0009	**0.001**^**a**^	0.0009	0.0007	0.0008
Methomyl	0.0025	0.0002	0.0005	0.0003	0.0002	0.0002	0.0002	0.0001	0.0001
Aldicarbfragment	0.001	0.0001	0.0003	0.0002	0.0001	0.0001	0.0001	9.4E-05	0.0001
Pirimicarb	0.035	0.0001	0.0003	0.0002	0.0001	0.0001	0.0001	9.4E-05	0.0001
Dioxacarb	0.001	**0.02**^**a**^	**0.06**^**a**^	**0.03**^**a**^	**0.02**^**a**^	**0.02**^**a**^	**0.02**^**a**^	**0.02**^**a**^	**0.02**^**a**^
Carbaryl	0.0075	3.0E-05	8.2E-05	4.7E-05	2.9E-05	3.3E-05	2.7E-05	2.3E-05	2.5E-05
Carbofuran	0.00015	5.9E-05	**0.0002**^**a**^	9.4E-05	5.8E-05	6.7E-05	5.4E-05	4.7E-05	5.1E-05
Alanycarb	0.001	**0.1**^**a**^	**0.3**^**a**^	**0.2**^**a**^	**0.1**^**a**^	**0.1**^**a**^	**0.1**^**a**^	**0.09**^**a**^	**0.1**^**a**^
Benfuracarb	0.01	**0.09**^**a**^	**0.2**^**a**^	**0.1**^**a**^	**0.09**^**a**^	**0.1**^**a**^	**0.08**^**a**^	**0.07**^**a**^	**0.08**^**a**^
Methiocarb	0.00025	0.0001	**0.0003**^**a**^	0.0002	0.0001	0.0001	0.0001	9.4E-05	0.0001
Imidacloprid	0.06	0.003	0.008	0.005	0.003	0.003	0.003	0.002	0.003
Acetamiprid	0.025	0.02	**0.06**^**a**^	**0.04**^**a**^	0.02	**0.03**^**a**^	0.02	0.02	0.02
Thiacloprid	0.01	1.2E-05	3.3E-05	1.9E-05	1.2E-05	1.3E-05	1.1E-05	9.4E-06	1.0E-05
Bifenthrin	0.015	0.0007	0.0015	0.001	0.0007	0.0008	0.0007	0.0006	0.0006
Lambda-Cyhalothrin	0.0012	0.001	**0.003**^**a**^	**0.002**^**a**^	0.001	0.001	0.0009	0.0008	0.0009
Deltamethrin	0.01	5.9E-05	0.0002	9.4E-05	5.8E-05	6.7E-05	5.4E-05	4.7E-05	5.1E-05
Cypermethrin	0.0016	**0.003**^**a**^	**0.008**^**a**^	**0.004**^**a**^	**0.003**^**a**^	**0.003**^**a**^	**0.002**^**a**^	**0.002**^**a**^	**0.002**^**a**^
Carbendazim	0.02	0.0003	0.0008	0.0005	0.0003	0.0003	0.0003	0.0002	0.0003
Imazalil	0.025	0.002	0.005	0.003	0.002	0.002	0.002	0.002	0.002
Metazachlor	0.08	0.0002	0.0005	0.0003	0.0002	0.0002	0.0002	0.0001	0.0002
Metalaxyl	0.08	5.9E-05	0.0002	9.4E-05	5.8E-05	6.7E-05	5.4E-05	4.7E-05	5.1E-05
Azaconazole	0.001	0.0002	0.0007	0.0004	0.0002	0.0003	0.0002	0.0002	0.0002
Clomazone	0.133	3.5E-06	9.9E-06	5.6E-06	3.5E-06	4E-06	3.3E-06	2.8E-06	3.0E-06
Azoxystrobin	0.2	0.01	0.04	0.02	0.01	0.01	0.01	0.01	0.01
Pyrimethanil	0.17	0.0006	0.002	0.0009	0.0006	0.0007	0.0005	0.0005	0.0005
Spirotetramat	0.05	0.0002	0.0005	0.0003	0.0002	0.0002	0.0002	0.0001	0.0002
Fenhexamid	0.2	0.1	**0.4**^**a**^	**0.2**^**a**^	0.1	0.2	0.1	0.1	0.1
Fenarimol	0.01	0.002	0.006	0.003	0.002	0.002	0.002	0.002	0.002
Fluazifop	0.004	**0.007**^**a**^	**0.02**^**a**^	**0.01**^**a**^	**0.007**^**a**^	**0.008**^**a**^	**0.006**^**a**^	**0.006**^**a**^	**0.006**^**a**^
Flufenoxuron	0.01	1.8E-05	4.9E-05	2.8E-05	1.7E-05	2E-05	1.6E-05	1.4E-05	1.5E-05
Pyriproxyfen	0.05	3.5E-06	9.9E-06	5.6E-06	3.5E-06	4E-06	3.3E-06	2.8E-06	3.0E-06
Quinoxyfen	0.2	0.0002	0.0005	0.0003	0.0002	0.0002	0.0002	0.0001	0.0002
Proquinazid	0.01	0.002	0.005	0.003	0.002	0.002	0.002	0.001	0.001

*BDL* Below detection limit, *ADI* Acceptable Daily Intake, *EDI* Estimated Daily Intake

^a^EDI greater than ADI (HQ > 1)

## Discussion

Pesticides were detected in all studied fruits and vegetables, with 39 active ingredients (AIs) detected in all samples and 18 AIs in at least some of the food samples. Fonofos, fenitrothion and fenhexamid concentrations were above the MRLs in watermelon, passion fruit, tomato, cabbage and eggplant. Risk assessment calculations show that EDIs for 18 pesticides were above the ADI in some cases, with HQs that ranged from 1 up to 443 and thus may pose chronic health risks. Children experienced the highest HQs and therefore potentially higher chronic health risks from pesticide residues in fruits and vegetables.

Overall, 29% of the pesticides we tested for had EDIs over an ADI. This is a high proportion of exceedances compared to other risk assessment studies (Szpyrka et al. 2013; Lozowicka et al. 2015; Mebdoua et al. 2017). When calculated by stage along the supply chain and age group, 16 and 18 pesticides respectively had high EDIs are above their ADI. As discussed by JA Vaccaro and FG Huffman (Vaccaro and Huffman 2017), age is a key dietary risk factor that should be considered while performing health risk assessment Several fruit and vegetable surveillance studies have estimated EDI and similar EDIs. Studies in Chile, Poland and Kazakhstan had EDIs ranging from < 0.001 to 5.2 (Lozowicka et al. 2015; Elgueta et al. 2017, 2019, 2020; Si et al. 2021; Szpyrka and Słowik-Borowiec 2019), which is within the range of our findings.

Many pesticides were detected in all studied fruits and vegetables with levels below the EU MRLs except for Fonofos, fenitrothion and fenhexamid. Our findings are consistent with existing literature showing detection of many pesticides in fruits and vegetables (Elgueta et al. 2019, 2020; Jallow et al. 2017; López-Dávila et al. 2021). Like our findings, many past studies have pesticide residue levels that are above MRL values, especially organophosphates like fenitrothion (Szpyrka et al. 2013; Mebdoua et al. 2017; Si et al. 2021; Szpyrka and Słowik-Borowiec 2019; Eslami et al. 2021; Kazar Soydan et al. 2021; Toptanci et al. 2021; Akoto et al. 2015). For example, recent studies in Ghana and Nigeria also found that many pesticides residue levels in produce were above the respective MRLs (Fosu et al. 2017; Adeleye et al. 2019a). The most frequently detected pesticides that have exceeded MRLs have been organophosphates, carbamates, pyrethroids and neonicotinoids based on studies in Uganda, Ghana, Egypt, Poland and Chile (Fuhrimann et al. 2021; Staudacher et al. 2020; Kaye et al. 2015; Atuhaire et al. 2017; Fosu et al. 2017; Issa et al. 2018; Szpyrka et al. 2013; Akomea-Frempong et al. 2017), especially in leafy vegetables (Elgueta et al. 2019; 2020). Given that MRLs are determined based on good agricultural practices (GAPs) in field experiments and not necessarily health risks (Fothergill and Abdelghani 2013; Salazar 2011), consumption of pesticides below the MRLs might exceed health-based exposure benchmarks depending on individual consumption patterns.

Our findings confirm similar findings to other studies carried out in Poland, Nigeria and Saudi Arabia which found that many pesticides had a HQ > 1 (Szpyrka et al. 2013; Odewale et al. 2021; Picó et al. 2018). On the other hand, literature from Turkey, Poland, Ghana, China and South Korea showed no chronic health risk associated with pesticide residues in fruits and vegetables (Si et al. 2021; Szpyrka and Słowik-Borowiec 2019; Kazar Soydan et al. 2021; Akoto et al. 2015; Szpyrka 2015; Park et al. 2021; Zhang et al. 2021; Yi et al. 2020). Using probabilistic modelling, Z Eslami, V Mahdavi and B Tajdar-Oranj (Eslami et al. 2021) in Iran found that pesticide residues did not pose health risks to adults and children. When assessed by stage along the supply chain, some pesticide showed a low HQ and consequently lower risk when consumed at farm than at other stages further along the supply chain, such as restaurants and homes. Our findings are similar to those from previous studies which have shown a higher chronic health risk for stages upstream along the chain (Akomea-Frempong et al. 2017; Jacxsens et al. 2017). When HQ was assessed by age, children more frequently experienced higher hazard quotients (18-13) compared with adults (11-9) with HQS up to 443, compared with a maximum HQ for adults at XX. Our findings are similar to findings from studies from Chile, Nigeria and China that assessed risk by age which found that chronic health risks were higher in children compared to adults (Elgueta et al. 2020; Si et al. 2021; Zhang et al. 2021; Adeleye et al. 2019b).

Our findings have implications on policy and future research. We used the EU MRLs and ADIs to evaluate exposures and risks, these benchmarks are lower and hence more sensitive than other guidelines. For example, Codex Alimentarius guidelines are higher, which would suggest lower health risks based on the exposure we evaluated. There is a need to develop Ugandan standards for MRLs and ADI based on local studies and context. The high HQs demonstrate in our study also demonstrate the need for routine monitoring and surveillance of pesticide residues in foods, especially in fruits and vegetables.

This study has several strengths and limitations. This study is the largest in Uganda to examine pesticide residues in fruits and vegetables; and we interviewed over 2000 residents to obtain information on dietary intake patterns. Dietary consumption data for fruit and vegetable was measured using a contextualised food album and thus presents a true reflection of the study community. We used mean residue concentrations to assess likely average exposures to consumers, but individual variability in eating patterns may result in higher or lower chronic exposures (Szpyrka et al. 2015). Additionally, we computed hazard quotients for consumption of individual foods. It is likely that consumers ate several different fruits or vegetables on any given day. In future analyses, we will use probabilistic methods to assess the range of potential exposures and health risks from more realistic diet patterns. We will also apply relative potency factors (RPFs) to assess cumulative health risks for pesticide classes with established RPFS (U.S. Environmental Protection Agency 2002). Fruits and vegetables were not tracked from farm to fork during sampling due cost and time challenges. Future studies examining pesticide residues along the farm to fork chain should track and sample individual produce lots from harvest to the consumer. Additionally, this study was carried out in a primarily urban community and may not represent a typical Ugandan rural setting. Finally, dietary consumption measurement did not cover the broad spectrum of fruits and vegetables but rather focussed on commonly consumed items within the study area (watermelon, passion fruit, tomato, cabbage and eggplant). However, the study area represents a large proportion of the Ugandan population and several commonly eaten foods.

## Conclusion

Sixty-two (62) pesticide residues were detected in fruits and vegetables from farm to fork. Concentrations of fonofos, fenitrothion and fenhexamid were above EU MRLs in watermelon, passion fruit, tomato, cabbages and eggplant. Exposures to 16 and 18 pesticides exceeded health-based benchmarks and potentially pose chronic health risks to consumers, especially to children. The study findings demonstrate the urgent need for routine pesticide monitoring and surveillance and risk assessment for fruits and vegetables in local Ugandan markets. There is also need to regulate the levels of pesticide in fruits and vegetables in order to protect consumers, especially the children who present higher chronic health risks.

## Supplementary Information

**Additional file 1: Table 1A.** Hazard quotient for pesticides with EDI greater than the ADI at different stages along the chain. This file contains pesticide that presented a high hazardous quotient at different stages along the chain from farm to fork that can potentially put the health of fruits and vegetable consumers at risk. **Table 2A.** Hazard quotient for pesticides with EDI greater than the ADI by age group. This file contains pesticide that presented a high hazardous quotient by age group that can potentially put the health of fruits and vegetable consumers at risk.

## Data Availability

The dataset used during the study is available from the corresponding author on reasonable request.

## Abbreviations

ADI: Acceptable Daily Intake
AIs: Active Ingredients
BDL: Below Detection Limits
BW: Body Weight
C: Mean concentration of each Pesticide
EDI: Estimated Daily Intake
EU MRLs: European Union Maximum Residual Limits
FVCR: Fruit and Vegetable Intake Rate
GAPs: Good Agricultural Practices
GC – MS: Gas Chromatography – Mass Spectrometry
HQ: Hazard Quotient
KMA: Kampala Metropolitan Area
LC – MS/MS: Liquid Chromatography – Tandem Mass Spectrometry
LOD: Limit of Detection
LOQ: Limit of Quantification
MRLs: Maximum Residual Limits
NCDs: Noncommunicable Diseases
QuEchERS: Quick, Easy, Cheap, Effective, Rugged and Safe
RPFs: Relative Potency Factors
U.S. FDA: United States Food and Drugs Authority
WHO: World Health Organisation
