# Supplementary material for: An assessment of health risks posed by consumption of pesticide residues in fruits and vegetables among residents in the Kampala Metropolitan Area in Uganda
Source: Int J Food Contam. 2022 Apr 28;9(1):4. doi: 10.1186/s40550-022-00090-9 (PMC9050770; doi:10.1186/s40550-022-00090-9)
Supplement: Supplementary file 1 — Additional file 1: Table 1A. Hazard quotient for pesticides with EDI greater than the ADI at different stages along the chain. This file contains pesticide that presented a high hazardous quotient at different stages along the chain from farm to fork that can potentially put the health of fruits and vegetable consumers at risk. Table 2A. Hazard quotient for pesticides with EDI greater than the ADI by age group. This file contains pesticide that presented a high hazardous quotient by age group that can potentially put the health of fruits and vegetable consumers at risk. [file 40550_2022_90_MOESM1_ESM.docx]

**Table A1: Hazard quotient for pesticides with EDI greater than the ADI at different stages along the chain**

| **Pesticides** | **Hazard Quotient (HQ)** | | | | |
| --- | --- | --- | --- | --- | --- |
|  | **Farm** | **Market** | **Street** | **Restaurant** | **Home** |
| Omethoate | 0.002 | 1.2 | BDL | 1.2 | 0.001 |
| Dimethoate | BDL | 1.5 | BDL | BDL | BDL |
| Fonofos | 27.5 | 29.5 | 0.2 | 39.3 | 13.8 |
| Dichlorvos | 442.6 | 7.4 | 4.4 | 22.1 | 36.9 |
| Quinalphos | 0.1 | 0.5 | 0.4 | 1.2 | 0.6 |
| Profenofos | 3.9 | 1.4 | 1.2 | 2.0 | 0.1 |
| Fenitrothion | 23.6 | 10.6 | 47.2 | 35.4 | 4.7 |
| Dioxacarb | 29.5 | 23.6 | 17.7 | BDL | BDL |
| Carbofuran | 0.03 | 0.004 | 2.0 | 1.6 | 0.002 |
| Alanycarb | 118.0 | 118.0 | 59.0 | 118.0 | 177.0 |
| Benfuracarb | 23.6 | 4.7 | 1.8 | 4.7E-11 | 1.8 |
| Acetamiprid | 1.7 | 0.9 | 0.5 | 0.5 | 0.2 |
| Cypermethrin | 2.6 | 1.1 | 3.0 | 0.7 | 0.4 |
| Fenhexamid | 0.6 | 0.9 | 1.8 | 0.03 | 0.2 |
| Fluazifop | 0.1 | 5.9 | BDL | BDL | BDL |

BDL – Below Detection Limit, HQ – Hazard Quotient

**Table A2: Hazard quotient for pesticides with EDI greater than the ADI by age group**

| **Pesticides** | **Hazard Quotient (HQ) by age group (years)** | | | | | | |
| --- | --- | --- | --- | --- | --- | --- | --- |
|  | **<5** | **05-12** | **13-19** | **20-24** | **25-35** | **36-49** | **50+** |
| Omethoate | 1.6 | 0.9 | 0.6 | 0.6 | 0.5 | 0.4 | 0.5 |
| Dimethoate | 1.2 | 0.7 | 0.4 | 0.5 | 0.4 | 0.3 | 0.4 |
| Fonofos | 68.2 | 38.8 | 24.0 | 27.6 | 22.5 | 19.4 | 20.9 |
| Dichlorvos | 444.3 | 253.1 | 156.5 | 180.2 | 146.5 | 126.5 | 136.2 |
| Quinalphos | 1.3 | 0.7 | 0.5 | 0.5 | 0.4 | 0.4 | 0.4 |
| Profenofos | 5.6 | 3.2 | 2.0 | 2.3 | 1.8 | 1.6 | 1.7 |
| Fenitrothion | 62.1 | 35.4 | 21.9 | 25.2 | 20.5 | 17.7 | 19.1 |
| Aminocarb | 2.6 | 1.5 | 0.9 | 1.1 | 0.9 | 0.7 | 0.8 |
| Dioxacarb | 55.1 | 31.4 | 19.4 | 22.4 | 18.2 | 15.7 | 16.9 |
| Carbofuran | 1.1 | 0.6 | 0.4 | 0.4 | 0.4 | 0.3 | 0.3 |
| Alanycarb | 314.3 | 179.0 | 110.74 | 127.5 | 103.6 | 89.5 | 96.4 |
| Benfuracarb | 24.8 | 14.1 | 8.7 | 10.1 | 8.2 | 7.1 | 7.6 |
| Methiocarb | 1.3 | 0.7 | 0.5 | 0.5 | 0.4 | 0.4 | 0.4 |
| Acetamiprid | 2.5 | 1.4 | 0.9 | 1.0 | 0.8 | 0.7 | 0.8 |
| Lambda-Cyhalothrin | 2.3 | 1.3 | 0.8 | 0.9 | 0.8 | 0.7 | 0.7 |
| Cypermethrin | 4.7 | 2.7 | 1.7 | 1.9 | 1.6 | 1.3 | 1.5 |
| Fenhexamid | 1.9 | 1.1 | 0.7 | 0.8 | 0.6 | 0.5 | 0.6 |
| Fluazifop | 4.6 | 2.8 | 1.7 | 2.0 | 1.6 | 1.4 | 1.5 |

HQ – Hazard Quotient
